# Precut over a pancreatic stent using the marking method to aid biliary cannulation in a patient with Roux-en-Y reconstruction

**DOI:** 10.1055/a-2268-2156

**Published:** 2024-03-01

**Authors:** Yuichi Takano, Naoki Tamai, Jun Noda, Tetsushi Azami, Fumitaka Niiya, Fumiya Nishimoto, Masatsugu Nagahama

**Affiliations:** 1 26858 Division of Gastroenterology, Department of Internal Medicine, Showa University Fujigaoka Hospital, Yokohama, Japan

Despite the remarkable development of balloon enteroscopy-assisted endoscopic retrograde cholangiopancreatography (ERCP), deep biliary cannulation in patients with surgically altered anatomy remains challenging 1 2 . We report a case where biliary cannulation was successfully achieved via precutting over a pancreatic stent using the marking method 3 .

A 78-year-old man with a history of total gastrectomy and Roux-en-Y reconstruction for gastric cancer visited our hospital with a chief complaint of epigastric pain. He was diagnosed with a common bile duct stone on computed tomography. A single-balloon enteroscope (SIF-H290S; Olympus Medical Systems, Tokyo, Japan) was inserted to reach the naïve papilla of Vater ( Fig. 1 **a** ). Wire-guided cannulation was attempted, but biliary cannulation was unsuccessful. It was possible to place a guidewire in the pancreatic duct, so a pancreatic guidewire-assisted technique was performed, but biliary cannulation was still impossible. A 5-Fr, 5-cm straight-type pancreatic stent (Geenen Pancreatic Stent Sets; Cook Meical Japan, Tokyo, Japan) was placed. Marking was performed using a needle knife (KD-10Q-1; Olympus Medical Systems) at the bulge of the papilla in the 6 o’clock direction from the pancreatic stent, which was believed to be the direction of the bile duct ( Fig. 1 **b** ). Fistulotomy was performed by making an incision between the pancreatic stent and the marking point. A red nodule was observed on the incised surface, suspected to be the biliary orifice ( Fig. 1 **c** ). The catheter was gently applied to the nodule, and deep biliary cannulation was achieved ( Fig. 1 **d** ). A 7-Fr, 7-cm double-pigtail biliary plastic stent was placed, and the procedure was completed without any adverse events ( Video 1 ). Endoscopic stone extraction was performed 5 days later.

**Fig. 1:**
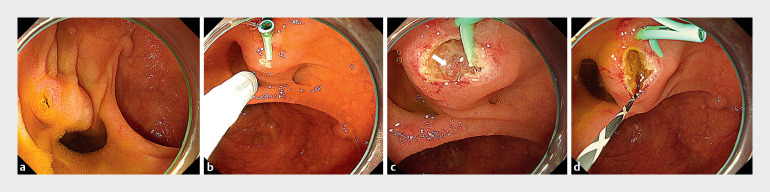
Endoscopic images showing: **a** the naïve papilla of Vater, which was reached with a single-balloon enteroscope; **b** a marking made using a needle knife at the bulge of the papilla in the 6 o’clock direction from the pancreatic stent, which was believed to be the direction of the bile duct; **c** a red nodule on the incised surface, suspected to be the biliary orifice (arrow), that was observed after fistulotomy had been performed by making an incision between the pancreatic stent and the marking point; **d** successful deep biliary cannulation after a catheter had been gently applied to the nodule.

Precutting is performed over a pancreatic stent using the marking method to aid biliary cannulation in a patient with Roux-en-Y reconstruction.Video 1

In patients with surgically altered anatomy, the papilla is upside down, making it difficult to recognize the direction of the bile duct 4 . Precutting in such patients requires a high degree of skill but, using the marking method, safe and reliable precutting can be performed.

Endoscopy_UCTN_Code_TTT_1AP_2AD

## Notes

Endoscopy E-Videos https://eref.thieme.de/e-videos
*E-Videos* is an open access online section of the journal *Endoscopy* , reporting on interesting cases and new techniques in gastroenterological endoscopy. All papers include a high-quality video and are published with a Creative Commons CC-BY license. Endoscopy E-Videos qualify for HINARI discounts and waivers and eligibility is automatically checked during the submission process. We grant 100% waivers to articles whose corresponding authors are based in Group A countries and 50% waivers to those who are based in Group B countries as classified by Research4Life (see: https://www.research4life.org/access/eligibility/ ). This section has its own submission website at https://mc.manuscriptcentral.com/e-videos .
